# Association between nutrient intake from vegetables and BMI category of in-school adolescents in urban and rural areas in Davao City, Philippines

**DOI:** 10.1016/j.dialog.2023.100116

**Published:** 2023-02-23

**Authors:** Kriza Faye A. Calumba, Miko Mariz C. Castro, Aileen Grace D. Delima, Melissa P. Loquias, Emma Ruth V. Bayogan, Pedro A. Alviola

**Affiliations:** a Department of Food Science and Chemistry, University of the Philippines Mindanao, Philippines; b School of Management, University of the Philippines Mindanao, Philippines; c Department of Biological Sciences and Environmental Studies, University of the Philippines Mindanao, Philippines

**Keywords:** Nutrient intake, Vegetable consumption, BMI, Adolescent diet, Nutrition

## Abstract

Consumption of vegetables may contribute to alleviating the double burden of malnutrition, which is widespread among adolescents. However, the link between specific nutrient intakes from vegetables and the body mass index (BMI) of in-school adolescents is not widely studied. This study determined the association between the nutrient intakes from the vegetables consumed and the BMI category of in-school adolescents in urban and rural areas in Davao City, Philippines. Self-reported vegetable consumption was collected from the respondents, and the corresponding nutrient intakes were calculated using the USDA food composition tables. The BMI of the participating adolescents was also measured. The results show that being underweight or overweight is generally associated with lower macronutrient and micronutrient intakes from the vegetables consumed, namely, bell pepper, bitter gourd, cabbage, carrot, chayote, Chinese cabbage, cucumber, eggplant, Malabar spinach, moringa, mung bean, okra, potato, sponge gourd, squash, string beans, sweet potato, sweet potato tops, taro, tomato, water spinach (*P* < 0.05). The nutrient intakes from vegetables consumed by adolescents from urban households were generally higher. The findings highlight the contribution of vegetables to the nutrient intakes in the adolescent population. This study reinforces the need for targeted dietary guidelines and further promotion of vegetables, especially indigenous ones, to improve the nutritional status of adolescents in the Philippines.

## 1 Introduction

Malnutrition has remained prevalent in low- and middle-income countries (LMIC), such as the Philippines. In a recent review, it was found that the number of undernourished individuals in Africa, Western Asia, and Oceania increased compared to ten years ago, while South Asia suffers the most from hidden hunger [1]. While several reports have focused on undernutrition, the incidence of overweight or obesity has also considerably increased. This double burden of malnutrition is widespread among adolescents (aged 10–19 years) in LMIC, where approximately 90% of the global adolescent population resides [[2], [3]]; however, the risk factors for this group remain neglected [[3], [4], [5]]. According to the Philippine National Nutrition Survey in 2013, 8.3% of adolescents were overweight or obese [6]. The prevalence of overweight and obese among adolescents increased from 4.9% in 2003 to 12% in 2018 [7]. In Davao City, Philippines, there was an 11% prevalence of overweight/obesity among adolescents, and those in non-poor households have a significantly higher prevalence than those in poor households [8]. As adolescence is a period of faster growth [9] and significant nutrition transition from childhood to adulthood, improper nutrition may lead to future health issues, affecting individual productivity in the labor market [10]. Nutritional status is positively associated with academic performance among adolescents; hence, malnutrition during this stage consequently hampers development on a global scale [11]. Therefore, there is a need for more extensive efforts to improve the nutritional practices of adolescents, primarily those residing in LMIC [2,4].

Diet plays a crucial role in ensuring the optimum growth and development of the adolescent population. Inadequacies in food and nutrient intakes can lead to impaired bodily functions and a higher risk of acquiring chronic non-communicable diseases [12], as well as viral, helminthic, parasitic, and malarial infections, especially among those in developing countries [3,13]. In a relevant study, the nutrient intakes of Filipino adolescents were shown to be inadequate in energy, fat, and key micronutrients [6]. Food consumption patterns have shifted to diets that are high in fat and insufficient in fruits and vegetables [6], and these habits can be possibly maintained into adulthood [9]. Since adolescence is a stage where individuals begin to make their own eating decisions, this provides a critical opportunity for introducing interventions that can influence attitudes toward healthy eating [3,14].

The Body Mass Index (BMI) is an accepted measure for categorizing individuals into different weight groups [15]. This method is equally or more important than total body fat measures [16] and is used to diagnose overweight and obesity among children and adolescents [17]. While BMI is arbitrary and subjective when categorizing groups, high BMI values have been consistently linked to increased morbidity and mortality. BMI can also set the standard for monitoring changes in the health of a population [18]. On the other hand, low BMI is especially detrimental to female adolescents entering motherhood [3]. A healthy diet was also associated with less obesity among 17-year-old adolescents. Diets play a role in inflammation and being overweight or obese, which are associated with mental health problems [19].

Disparities in nutritional status across populations can be influenced by socioeconomic status and place of residence. Food options are generally more widely accessible and available in urban than rural areas [6]. A higher percentage of Filipino adolescents manifesting nutrient inadequacies was observed in rural areas, where food choices are typically limited [20]. Adolescents residing in urban areas in the Philippines had a low vegetable consumption, similar to other Southeast Asian countries [21]. Urban adolescents were also shown to consume more snacks, which was associated with a higher BMI [22]. Moreover, studies have established the lower consumption of fruits and vegetables in the Philippines compared to other countries. Low intake of these food groups is largely associated with a higher risk of coronary heart disease and ischaemic stroke [9,20,23]. Filipino adolescents consumed fewer servings of vegetables than recommended [24].

Indigenous vegetables refer to plants grown in their centers of origin or crops whose natural home is in a specified region [[25], [26]]. In the Philippines, indigenous vegetables can provide abundant nutrients at a lower cost and effort than more popular vegetables available in the market [27]. While some indigenous vegetables in the country exhibit antioxidant activity [28], these underutilized vegetables have not been thoroughly explored. For example, Keatinge et al. [25] emphasized the need for further promotion of the contribution of indigenous vegetables to the nutritional health of various populations. Utilizing indigenous vegetables has great potential in combating food insecurity in the country [27]. The general objective of this study was to determine the association between the nutrient intakes from the vegetables consumed and the BMI category of in-school adolescents in urban and rural areas in Davao City, Philippines. The schools selected are representative of the urban and rural areas in Davao City. The sample schools were partitioned into urban and rural areas due to documented disparities in health and nutrition outcomes. As studies have shown, disparities in socioeconomic status and differential access to healthcare and medicine have disadvantaged children residing in rural areas by having relatively poor nutritional intakes compared to those living in urban areas [[29], [30]]. This is especially important given that the current pandemic and future health outbreaks magnify children's vulnerabilities, and there is a need to improve nutrition practices to prevent adverse health outcomes in children and adolescents.

## 2 Materials and methods

### 2.1 Study design and participants

This study used power analysis to calculate the sample size. In estimating the required sample size, the following specifications were used: power = 0.6, alpha error = 0.05, and an assumption of medium effect size Cohen's d = 0.5. The effect size is crucial in estimating the sample size as it reports the magnitude of the effects in a quantitative study. Hence, this complements the *P*-value approach, which reports whether a statistical significance exists [31]. The effect size was based on Cohen's method for paired samples, where an effect size of 0.3 was considered small, 0.5 medium, and 0.8 large [[32], [33], [34]]. Using these specifications, the minimum sample size of 68 was calculated, with 23 samples for group 1 and 45 samples for group 2. The computation of the total sample size was done using the G*Power version 3.1.9.4 sample size calculator [35]. We also note from the central limit theorem, that as sample size increases (*n* ≥ 30), inferences become reliable because the sample distribution approaches normality [[36], [37]].

A stratified random sampling was employed. The strata, in this case, the barangays, were identified based on the classification (urban or rural) by the Office of the City Planning and Development Coordinator in Davao City, Philippines [38]. After identifying the barangays, random selection was done on public high schools in urban and rural areas in Davao City, Philippines. Three schools from each category were identified. These include Elias B. Lopez Memorial National High School, Sto. Nino National High School, and Francisco Bangoy National High School in the urban classification, and Waan National High School, Suawan National High School, and Emilio Estipona National High School in the rural category. The number of students per school was determined using proportional allocation based on the 2018–2019 enrollment data secured from the Department of Education, Davao City Division. Participants were eleventh-grade students with a mean age of 17 years old. The Food and Nutrition Research Institute (FNRI) [7] defines adolescents as individuals belonging to the age group 10 to 19 years.

### 2.2 Survey instrument

The survey determined the participants' household socio-demographic characteristics and vegetable consumption (including indigenous vegetables). These include data on sex, age, household size, livelihood, and self-reported food intakes in three days. In gathering the information on food including vegetable intake, a 3-day food record was used. Compared with other food frequency assessments such as 24-h or 5-day food frequency, this approach has been judged as better and more accurate [[39], [40]]. Using the food composition table developed by the United States Department of Agriculture [41], each vegetable intake was calculated for energy (kcal), protein (g), lipid (g), carbohydrate by difference (g), dietary fiber (g), calcium, iron, magnesium, phosphorus, potassium, sodium, zinc, vitamin C, thiamin, riboflavin, niacin, vitamin B6, folate, vitamin A (retinol activity equivalent or RAE), vitamin E, and vitamin K, wherein all vitamins and minerals were calculated in mg [[42], [43]]. Using a guided open-ended questionnaire, the participants were also asked to describe their perceptions and practices toward eating vegetables. The responses were grouped and tabulated.

### 2.3 Data collection

Data were collected through personal interviews using a pre-tested questionnaire. The questionnaire was translated into the local dialect. The study was conducted following the protocols set by the Declaration of Helsinki [44], where the research objective was thoroughly explained and all respondents provided written informed consent before their participation. For adolescent respondents less than 18 years old, written consent was obtained from the parents/guardians. Data collection was conducted with approval from the Office of Research at the University of the Philippines Mindanao. Data were appropriately managed to ensure privacy and anonymity.

### 2.4 Body mass index

Body mass index (BMI) is a simple and convenient way to assess the appropriateness of the weight of adolescents [18,45]. The BMI was obtained from the height and weight assessed by trained enumerators. The BMI of each participant was calculated as weight in kg divided by height in square meters [46]. Participants with BMI higher than 25 were classified as overweight, <18.5 were underweight, and those with BMI within 18.5–24.9 were categorized under normal weight [[47], [48]].

### 2.5 Data analysis

**Fig. 1:**
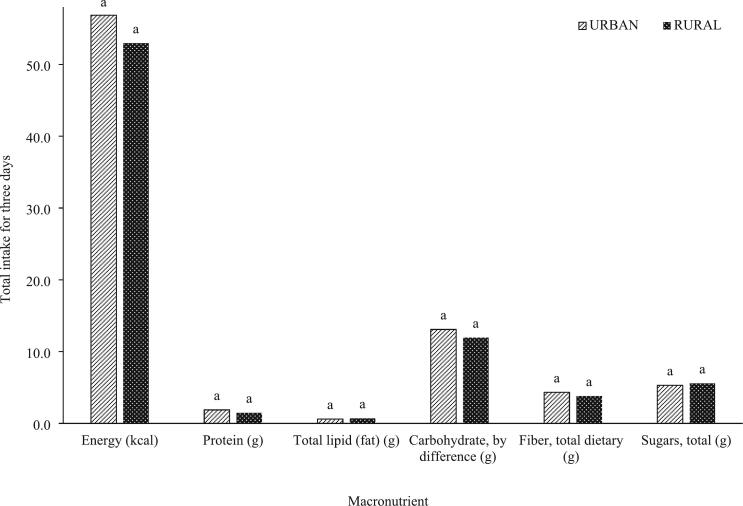
Macronutrient intakes from indigenous vegetables consumed by in-school adolescents (*N* = 69) in urban vs. rural areas in a span of three days. ^a^No significant differences were observed (*P* > 0.05).

**Fig. 2:**
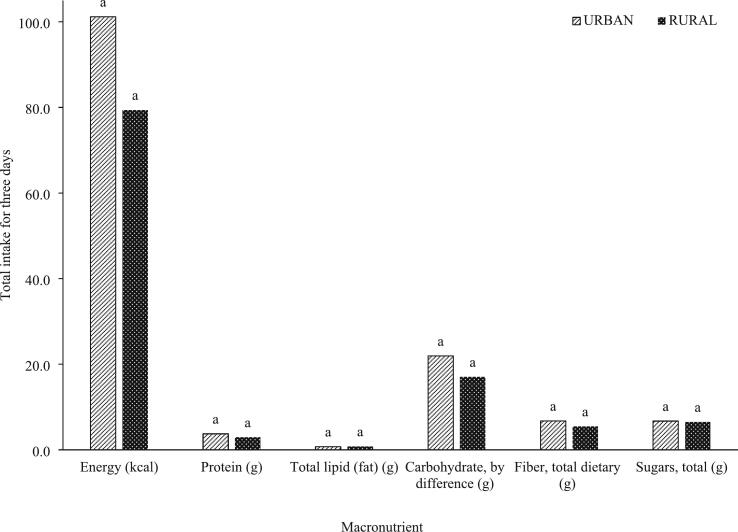
Macronutrient intakes from all vegetables consumed by in-school adolescents (*N* = 69) in urban vs. rural areas in a span of three days. ^a^No significant differences were observed (*P* > 0.05).

**Fig. 3:**
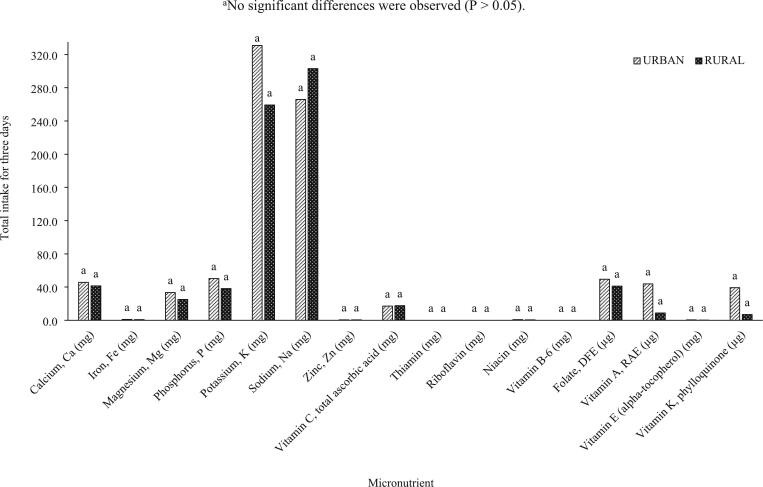
Micronutrient intakes from indigenous vegetables consumed by in-school adolescents (N = 69) in urban vs. rural areas in a span of three days. ^a^No significant differences were observed (P > 0.05).

**Fig. 4:**
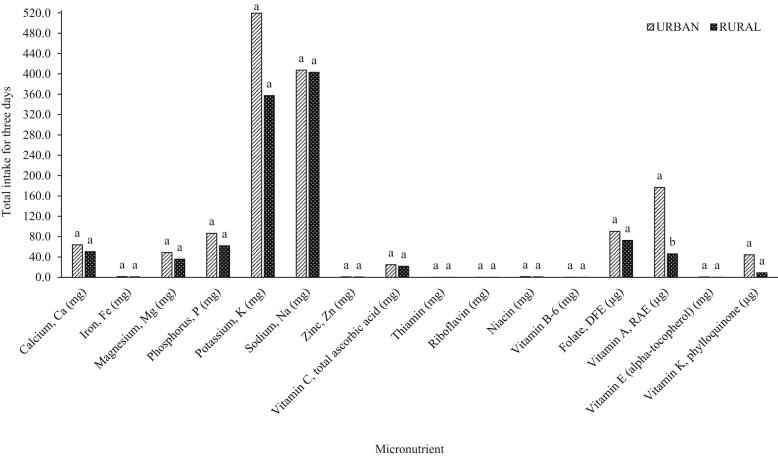
Micronutrient intakes from all vegetables consumed by in-school adolescents (N = 69) in urban vs. rural areas in a span of three days. ^a^Means with different letters within the same nutrient are significantly different at *P* < 0.05.

All returned questionnaires were checked for completeness to exclude missing data. The mean macronutrient and micronutrient intakes from the vegetables were obtained. Using SPSS version 27, an independent *t*-test was used to assess differences between nutrient intakes in urban and rural areas at a 0.05 level of significance (Fig. 1, Fig. 2, Fig. 3, Fig. 4). To evaluate the association with BMI, a test of homogeneity of variances was first conducted. For nutrients with a *p-*value less than 0.05 for the homogeneity, indicating unequal variances, Welch's test was used to determine significant differences among intakes across the three BMI categories. Welch's test is recommended for unequal variances [[49], [50]]. Otherwise, a one-way analysis of variance was employed to determine differences in the results among the BMI categories [46]. The Games-Howell test [49] and the Tukey HSD test were used as post-hoc for unequal and equal variances, respectively (Table 2).

## 3 Results

### 3.1 Socio-demographic characteristics

As presented in Table 1, 43% of the respondents were male and 57% were female. The majority of the adolescents (68%) were less than 18 years old. More than half of the rural (67%) participants and urban (60%) areas had 4 to 6 members in the household with income sources mostly from non-farming activities.

**Table 1:** Socio-demographic profile of the respondents.

Variable		Classification				Total	
		Rural		Urban			
		*N* = 24	Percent	*N* = 45	Percent	N = 69	Percent
Sex	Male	11	46	19	42	30	43
	Female	13	54	26	58	39	57
Age	18 and above	5	21	17	38	22	32
	less than 18	19	79	28	62	47	68
Household size	1–3 members	1	4.2	6	13	7	10
	4–6 members	16	67	27	60	43	62
	7–9 members	6	25	6	13	12	17
	10 and above members	1	4.2	6	13	7	10
Livelihood	Farming	8	38	8	18	16	23
	Non-Farming	13	62	36	82	49	71
	Both	3	13	1	2.2	4	6

### 3.2 Macronutrient and micronutrient intakes from vegetables

In this study, the results obtained for macronutrient intake had a generally similar trend reported in several studies where rural households have lower nutrient inadequacies, although no significant differences were mainly observed for indigenous vegetables (Fig. 1) and total vegetables (Fig. 2). The micronutrient intakes from vegetables consumed by adolescents from urban households were generally higher (Fig. 3, Fig. 4) except for sodium obtained from indigenous vegetables (265.82 mg for urban; 302.92 mg for rural), although these two values are not significantly different. While the macronutrient intakes from indigenous vegetables of both urban and rural groups are not different (Fig. 3), Fig. 4 shows that the vitamin A intake from all vegetables consumed by urban adolescents was significantly higher (176.65 μg RAE) compared to the rural classification (46.26 μg RAE). The indigenous vegetables considered in this study are bitter gourd, chayote, cucumber, eggplant, Malabar spinach, moringa, okra, sponge gourd, squash, string beans, sweet potato, sweet potato tops, taro, and water spinach.

### 3.3 Association between nutrient intakes from vegetables and BMI category

This study examined the association between vegetable consumption and BMI category in adolescents. Table 2 presents the mean nutrient intakes from indigenous and total vegetables and their association with the BMI categories classified as underweight (<18.5), normal (18.5–24.9), and overweight (> 25) [[47], [48]]. The intakes of macronutrients and micronutrients obtained from indigenous vegetables were not significantly different among the BMI categories, except for vitamin C, whose intake was higher in the normal BMI category than in the overweight group.

**Table 2:** Association between nutrient intakes from vegetables and BMI category of in-school adolescents (*N* = 69).

Nutrient	BMI Category^1^	Mean Intake from Indigenous Vegetables^2^	Mean Intake from All Vegetables^3^
Energy (kcal)	Underweight	36.45^a^	54.66^B^
	Normal	71.81^a^	127.40^A^
	Overweight	32.29^a^	43.63^B^
Protein (g)	Underweight	1.04^a^	1.69^B^
	Normal	2.30^a^	4.99^A^
	Overweight	1.11^a^	1.29^B^
Total lipid (fat) (g)	Underweight	0.50^a^	0.55^A^
	Normal	0.81^a^	1.00^A^
	Overweight	0.29^a^	0.32^A^
Carbohydrate, by difference (g)	Underweight	8.20^a^	12.18^B^
	Normal	16.45^a^	27.36^A^
	Overweight	7.68^a^	9.14^B^
Fiber, total dietary (g)	Underweight	2.65^a^	3.46^B^
	Normal	5.41^a^	8.73^A^
	Overweight	2.41^a^	2.98^B^
Sugars, total (g)	Underweight	3.67^a^	4.12^A^
	Normal	6.91^a^	8.80^A^
	Overweight	3.12^a^	3.78^A^
Calcium, Ca (mg)	Underweight	20.50^a^	26.21^B^
	Normal	63.43^a^	85.70^A^
	Overweight	22.00^a^	28.63^B^
Iron, Fe (mg)	Underweight	0.50^a^	0.64^B^
	Normal	1.43^a^	2.02^A^
	Overweight	0.59^a^	0.67^B^
Magnesium, Mg (mg)	Underweight	17.46^a^	23.25^B^
	Normal	41.13^a^	61.67^A^
	Overweight	18.29^a^	20.50^AB^
Phosphorus, P (mg)	Underweight	27.25^a^	40.63^B^
	Normal	61.61^a^	109.98^A^
	Overweight	26.57^a^	32.28^B^
Potassium, K (mg)	Underweight	200.09^a^	281.38^B^
	Normal	399.46^a^	625.55^A^
	Overweight	160.86^a^	202.89^B^
Sodium, Na (mg)	Underweight	134.88^a^	166.13^B^
	Normal	397.63^a^	607.10^A^
	Overweight	126.43^a^	135.33^B^
Zinc, Zn (mg)	Underweight	0.39^a^	0.48^B^
	Normal	0.86^a^	l.21^A^
	Overweight	0.25^a^	0.29^B^
Vitamin C, total ascorbic acid (mg)	Underweight	11.83^ab^	14.27^B^
	Normal	23.12^a^	32.89^A^
	Overweight	4.17^b^	7.06^B^
Thiamin (mg)	Underweight	0.06^a^	0.08^B^
	Normal	0.10^a^	0.18^A^
	Overweight	0.07^a^	0.08^AB^
Riboflavin (mg)	Underweight	0.04^a^	0.05^B^
	Normal	0.08^a^	0.12^A^
	Overweight	0.03^a^	0.04^AB^
Niacin (mg)	Underweight	0.52^a^	0.78^A^
	Normal	1.00^a^	1.61^A^
	Overweight	0.60^a^	0.70^A^
Vitamin B-6 (mg)	Underweight	0.09^a^	0.14^B^
	Normal	0.17^a^	0.30^A^
	Overweight	0.08^a^	0.11^AB^
Folate, DFE (μg)	Underweight	28.54^a^	39.92^B^
	Normal	63.04^a^	123.24^A^
	Overweight	20.00^a^	24.54^B^
Vitamin A, RAE (μg)	Underweight	8.17^a^	60.08^A^
	Normal	51.05^a^	178.20^A^
	Overweight	8.50^a^	120.83^A^
Vitamin E (alpha-tocopherol) (mg)	Underweight	0.31^a^	0.39^A^
	Normal	0.53^a^	0.74^A^
	Overweight	0.30^a^	0.44^A^
Vitamin K, phylloquinone (μg)	Underweight	6.33^a^	7.57^A^
	Normal	46.14^a^	51.47^A^
	Overweight	3.76^a^	10.56^A^

a-b Means with no common letter within the same nutrient from indigenous vegetables across BMI categories are significantly different (*P* < 0.05).

A-B Means with no common letter within the same nutrient from all vegetables across BMI categories are significantly different (*P* < 0.05).

1 Underweight (*N* = 24), normal (*N* = 38), overweight (*N* = 7).

2 Bitter gourd, chayote, cucumber, eggplant, Malabar spinach, moringa, okra, sponge gourd, squash, string beans, sweet potato, sweet potato tops, taro, and water spinach.

3 Bell pepper, bitter gourd, cabbage, carrot, chayote, Chinese cabbage, cucumber, eggplant, Malabar spinach, moringa, mung bean, okra, potato, sponge gourd, squash, string beans, sweet potato, sweet potato tops, taro, tomato, water spinach.

For the total vegetables, the mean intakes of energy, protein, carbohydrate, total dietary fiber, and most micronutrients, namely calcium, iron, phosphorus, potassium, sodium, zinc, vitamin C, and folate, are significantly higher in the normal BMI category compared to the underweight and overweight categories (Table 2). The intakes of magnesium, thiamin, riboflavin, and vitamin B-6 from all vegetables were significantly higher in the normal BMI than in the underweight group.

### 3.4 Perceptions on vegetable consumption

As listed in Table 3, 29% of the responses state that vegetables contribute to a good body appearance, while 28% of the responses consider vegetables as good-tasting and 22% mention that vegetables are healthy and nutritious.

**Table 3:** Perceptions of vegetables among in-school adolescents based on multiple responses (*N* = 69).

Perception	Percent responses
Good for body appearance/ for a fit body/ for a fresh body feeling	29
Taste good	28
Healthy/ nutritious/ provide energy	22
Affordable	5
Juicy/ sweet	4
Increase resistance against diseases	3
Substitute for vitamins	2
Favorite/ like eating vegetables	2
Only food choice when hungry	2
Only when feeling like eating	1
Do not like sweet food/taste	2
Total	100

Perceptions listed above have been translated from the local dialect.

## 4 Discussion

The low vegetable consumption of Filipino adolescents can result in the poor intake of fiber and other nutrients, which may cause slow growth rates, inadequate bone mass, and impaired cognitive functioning [6]. Insufficient vegetable intake was described to be common in rural households where a lower prevalence of nutritional adequacy was observed [6]; however, no significant differences were generally observed. Malabar spinach, which contains vitamin A [51] was more often consumed by urban participants, which may have resulted in a significantly higher intake of this vitamin. The abundant nutrients in the indigenous vegetables are listed in Table 4, while the nutritional profile [41] of the indigenous and non-indigenous vegetables included in the study is presented in Table 5. On average, the indigenous vegetables have higher content of the minerals calcium, iron, magnesium, and zinc, as well as higher riboflavin, vitamin E, and vitamin K compared to the non-indigenous vegetables in the current study.

**Table 4:** Abundant nutrients in the indigenous vegetables studied.

Vegetable	Abundant nutrients	References
Bitter gourd (*Momordia* *charantia* L.)	Protein, vitamin C, folic acid, calcium, phosphorus, sodium, manganese, copper, and zinc (predominant in the light green big type)	[52]
Chayote (*Sechium* *edule* (Jacq.) Sw.)	Fiber, potassium, calcium, phosphorus, and magnesium	[53]
Cucumber (*Cucumis* *sativus* L.)	Vitamin C (higher in the rind than the pulp)	[54]
Eggplant (*Solanum* *melongena* L.)	Minerals, vitamins, fiber, protein, antioxidants, phytochemicals	[55]
Malabar spinach (*Basella* *alba* L.)	Vitamin A	[51]
Water spinach (*Ipomoea* *aquatica* Forssk.)	Protein, calcium, vitamin A, and vitamin C	[56]
Moringa (*Moringa* *oleifera* Lam.)	Calcium and total phenolic content	[57]
Taro (*Colocasia* *esculenta* (L.) Schott)	Fiber	[57]
Okra (*Abelmoschus* *esculentus* (L.) Moench)	Digestible carbohydrate	[57]
Sponge gourd (*Luffa* *acutangula* (L.) Roxb.)	Crude fiber	[58]
Squash (*Cucurbita* *maxima* Duchesne)	Vitamin C	[59]
String beans (*Phaseolus* *vulgaris* L.)	Protein, carbohydrates, vitamins, minerals, and unsaturated fatty acids	[60]
Sweet potato (*Ipomoea* *batatas* (L.) Lam)	β-carotene, magnesium, iron, copper, manganese, calcium, potassium), vita- mins (B1, B6, C, E), and dietary fiber	[62]
Sweet potato (*Ipomoea* *batatas* (L.) Lam) leaves	Lutein, dietary fiber, potassium, calcium, phosphorus, magnesium, sodium, vitamin C, vitamin E, and B-complex vitamins	[[61], [62]]

**Table 5:** Average nutritional profile of the vegetables studied.

	Indigenous vegetables^1^	Non-indigenous vegetables^2^
Macronutrient		
Energy (kcal)	27.01	39.53
Protein (g)	0.97	1.66
Total lipid (fat) (g)	0.27	0.12
Carbohydrate, by difference (g)	5.92	7.88
Fiber, total dietary (g)	1.74	2.10
Sugars, total (g)	1.88	1.64
Minerals (mg)		
Calcium, Ca	28.61	14.60
Iron, Fe	0.65	0.38
Magnesium, Mg	17.73	13.11
Phosphorus, P	23.34	31.28
Potassium, K	153.49	159.73
Sodium, Na	103.07	184.31
Zinc, Zn	0.31	0.22
Vitamins		
Vitamin C, total ascorbic acid (mg)	7.95	9.10
Thiamin (mg)	0.04	0.05
Riboflavin (mg)	0.05	0.02
Niacin (mg)	0.37	0.44
Vitamin B-6 (mg)	0.06	0.10
Folate, DFE (μg)	26.68	36.13
Vitamin A, RAE (μg)	47.18	66.16
Vitamin A (IU)	1104.50	1325.50
Vitamin E (alpha-tocopherol) (mg)	0.28	0.14
Vitamin K, phylloquinone (μg)	43.03	4.30

1 Bitter gourd, chayote, cucumber, eggplant, Malabar spinach, moringa, okra, sponge gourd, squash, string beans, sweet potato, sweet potato tops, taro, and water spinach.

2 Bell pepper, cabbage, carrot, Chinese cabbage, mung bean, potato, tomato.

The intake of vegetables is affected by socio-environmental and socioeconomic factors, such as a family environment that is supportive of vegetable consumption, adequate nutrition education, and affordability. The relatively lower vegetable intake among junior and senior high school students was associated with poor knowledge about what and how much they should eat [9]. Smartphone addiction [63] and nomophobia (no mobile phone phobia) were also recently identified as significant predictors of nutrition, along with low vegetable intake, among Filipino adolescents [64]. A systematic review explains the positive association between the consumption of green and yellow vegetables and mental health [65]. It is also stated that only about half of the sampled Filipino students, majority of which were 17 years old, consume dark green leafy vegetables frequently or often [66]. An effective nutrition curriculum can also largely improve exposure to vegetables and has the potential in increasing vegetable consumption and corresponding nutrient intakes among students [67], including in-school adolescents as described in the present study. Affordability is another crucial factor, as it was shown to influence vegetable consumption, especially among low-income households [68].

The results generally describe the association between high nutrient intakes from vegetables and having a normal BMI. These observations are consistent with one study which stated that the probability of having a BMI above 25 increases when consumption of vegetables is low [[69], [70]], suggesting that a high intake of vegetables helps maintain optimal weight and decrease the risk of developing diet-related diseases such as hypertension, hyperlipidemia, and diabetes [45]. Additionally, areas experiencing diminished access to vegetables such as food deserts can impact the nutritional status of an individual [71], and these food deserts are linked with increasing BMI [72].

Moreover, low affordability of vegetables as well as the emergence of fast-foods with high glycemic indexes and fats could be factors contributing to low vegetable intake and consequently a high BMI [71,73]. More frequent cooking and meal planning were also associated with greater vegetable intake and lower BMI among first-year college students [74]. Likewise, participants who reported a lower intake of vegetables belonged to the higher BMI category [9]. The results support dietary recommendations on the regular consumption of vegetables to maintain a healthy weight and good nutrition. Indigenous vegetables, which are widely available in the country but underutilized, must also be promoted.

Filipino adolescents were previously described to view vegetables as being good for their health [24]. Interestingly, only 5% of the responses state that vegetables are affordable. The consumption of vegetables decreases as they become less affordable [73]. The affordability of vegetables can be increased through incentive programs that could facilitate more vegetable consumption especially among low-income individuals, although these do not directly address the cost problem [75]. Further, in order to lower prices of indigenous vegetables, broad policy interventions such as reducing the transport cost by providing access to better roads, improving productivity, and decreasing post-harvest losses are needed, [[76], [77], [78]]. Moreover, the highest proportion of respondents from both urban (59%) and rural (27%) areas eat vegetables at home than in other places (e.g., school, market, farm). Availability of vegetables at home has been positively associated with vegetable intake, highlighting the important role of parents in ensuring sufficient vegetable intakes among children and adolescents [79]. In one study, low vegetable intake is more likely among children with obese parents [80]. The present survey also found that 43% of the responding adolescents from urban households eat fewer or the same amounts of vegetables now than before. These results are contrary to those obtained by Albani et al. [81], where vegetable intake did not change with age. Nonetheless, adolescents are more independent and have greater control over their diet preferences, so intervening during this stage can create a significant and positive impact on their nutrition [70].

## 5 Conclusions and recommendations

This study determined the association between the nutrient intakes from vegetables and the BMI of in-school adolescents in urban and rural areas in Davao City, Philippines. Being overweight or underweight is generally associated with lower macronutrient and micronutrient intakes from the vegetables consumed (*P* < 0.05). Micronutrient-deficient in-school adolescents are highly prone to develop iron deficiency, poor vitamin A status, and anemia-related disorders. Consequently, sustained exposure to these diseases impacts children particularly adolescent academic performance, which in turn affects their labor productivity as they become adults.

The findings of this study can serve as a basis for further improving dietary guidelines for adolescents, particularly in incorporating vegetables, especially indigenous ones, in the diet to maintain a normal BMI. Indigenous vegetables need to be widely promoted through home gardening, food product development, reinforced consumption in the family, and participation in school and community vegetable gardening programs. The affordability of vegetables is also a significant factor that can increase overall vegetable consumption. Also, the inclusion of the importance of vegetable consumption, particularly in cooking class curricula, may nudge further adolescent vegetable consumption. Moreover, in designing such interventions to increase the vegetable consumption of adolescents, focused efforts must be made in understanding their food habits and preferences. Through this, the nutrient intake disparity from eating vegetables and the recommended intakes among adolescents may be considerably lessened. Moreover, in-school adolescents in rural areas, in particular, are recommended to consume more vitamin A from vegetables. Regular consumption of vegetables may help prevent the double burden of malnutrition in both urban and rural areas, especially during a health crisis where nutritional status may be more compromised.

However, the results of this study are limited to the food intake provided by the adolescents, and under-or over-reporting of vegetables may be possible due to varying perceptions of portion sizes. The results of this study also cannot be generalized to other cities. Moreover, the nutritional status of the participants was assessed using only BMI as the variable. Lastly, while an association has been established between nutrient intake from vegetables and the BMI category of the study participants, this does not allow for any causal inferences. Weight gain is a chronic process that could be influenced by long-term dietary intake. It is recommended that the long-term effect of a putative cause, such as nutrient intake from vegetables, on BMI levels be explored in future studies.

## Funding statement

This study was funded by the University of the Philippines Mindanao 2019 In-house Research Grant.

## Author's contribution

KFAC, PAA, and ERVB planned and designed the study. All authors participated in the data collection. KFAC and MMCC analyzed and interpreted the data. KFAC wrote the manuscript. All authors reviewed and approved the final manuscript.

## Declaration of Competing Interest

The authors declare that they have no conflict of interest.
